# The emerging SARS-CoV, MERS-CoV, and SARS-CoV-2: An insight into the viruses zoonotic aspects

**DOI:** 10.14202/vetworld.2021.190-199

**Published:** 2021-01-23

**Authors:** Karima A. Al-Salihi, Jenan Mahmood Khalaf

**Affiliations:** 1 Department of Internal Medicine and Zoonotic Diseases, College of Veterinary Medicine, Al-Muthanna University, Al-Muthanna Province, Iraq; 2 Department of Internal and Preventive Medicine, Unit of Zoonotic Diseases, College of Veterinary Medicine, University of Baghdad, Baghdad, Iraq

**Keywords:** Bats, coronavirus disease-19, pneumonia, RNA viruses, zoonotic

## Abstract

Zoonotic coronavirus disease (COVID) has emerged in the past two decades and caused a pandemic that has produced a significant universal health alarm. Severe acute respiratory syndrome coronavirus (SARS-CoV) and Middle East respiratory syndrome-CoV (MERS-CoV) emerged in 2002 and 2012, respectively, provoking severe lower respiratory infection and deadly pneumonia. COVID-19 is a severe respiratory disease caused by the new strain of novel CoV (SARS-CoV-2). The zoonotic aspects of the SARS-CoV-2 in comparison to SARS-CoV and MERS-CoV are highlighted in this article. COVID-19 has rapidly become a pandemic and has spread and infected millions of people worldwide. As of November 19, 2020, the date of submitting this review, the total CoV cases, deaths, and recovered patients are 56,828,218, 1,359,320, and 39,548,923, respectively. In conclusion, COVID-19 has particularly altered the opinion of the significance of zoonotic diseases and their animal origins and the intermediate reservoirs, which may be unknown wild animals. Genetically, the SARS-CoV-2 is related to the SARS-like bat CoVs and shares 85% identity with the SARS-CoV that is derived from the SARS-like bat CoVs. However, the virus is related to a lesser extent to the MERS-CoV. The SARS-CoV-2 uses the same receptor-binding domain receptor of the SARS-CoV – the angiotensin-converting enzyme 2; conversely, DPP4 (CD26). It has not been proved that the MERS-CoVs primary receptor is the receptor of the SARS-CoV-2.

## Introduction

During the past decades, the zoonotic coronavirus disease (COVID) has emerged worldwide and caused fatal human diseases. These diseases are severe acute respiratory syndrome (SARS), Middle East respiratory syndrome (MERS), and COVID-19, which have led to fatal pandemic outbreaks, posing an actual danger to public health around the world [1]. The first defining human coronavirus (CoV) has been from patients with a common cold, in the 1960s. A diversity of pathological conditions has been reported in domestic animals due to CoV in the early 1970s [2]. Since the 1960s, CoVs have been reported to be causative agents of severe diseases in different animal species, including pet animals. The research and investigations on animal CoVs have been well developed during the 20^th^ century [3-5]. Various degrees of respiratory infections caused by CoVs have been reported in bovines and rats. CoV is also the cause of infectious bronchitis in chickens, which produces a severe economic loss in poultry manufacturers in different countries [6]. The CoVs belong to the *Coronaviridae* family, which are single-stranded RNA viruses.

Moreover, CoVs have a positive-sense RNA genome that ranges in size from 26 to 32 kilobases. The CoV membrane appeared like a crown under electron microscopy, which formed a famous feature of the virus – “the crown” – called “corona” in Latin [7-9]. The CoVs have owned the same organizational structures, including 16 non-structural proteins, the open reading frame (ORF) 1a/b at the 5’ end, surrounded by structural protein spike (S), envelope (E), and membrane (M). Furthermore, there is a nucleocapsid (N) that is encoded by other ORFs at the 3’ end. In general, there are four genera of CoVs according to the phylogenic classification. These genera are Groups 1, 2, 3, and 4, which are designated as alpha-CoV, beta-CoV, gamma-CoV, and delta-CoV, respectively. The beta-CoV is comprised four lineages, including A, B, C, and D, while hemagglutinin esterase is a smaller protein, encoded within lineage A viruses, and works similar to the S protein [10].

Until 2002, CoVs had been considered as minor pathogens for humans, and the 229E, OC43, NL63, and HKU1 were the causative agents for the typical common cold symptoms that revealed severe infection in older and immunocompetent people, infants, children, and young. However, in general, these viruses caused a self-limiting mild respiratory infection [11]. Although, this perception completely changed with the emergence of highly pathogenic diseases such as SARS and MERS. Both these diseases were zoonotic in origin and have been associated with fatal sickness [12-21]. In December 2019, novel CoV SARS-CoV-2 was diagnosed from patients in China/Wuhan. The sick people suffered from an unknown cause of pneumonia. Later on, this disease was named CoVID-2019 and characterized by the rapid distribution of outbreaks worldwide [22].

In addition, the undetected sources of transmission have been suggested, by an epidemiological study, to be from the Wuhan seafood market [23]. The pilot report based on the molecular analysis suggested that snakes were the possible origin of SARS-CoV-2 [24]. However, the source of infection and specific relation with animal hosts have not been recognized yet, and the recent agreement supports the theory of association of mammalians or birds. The molecular investigation has suggested that SARS-CoV-2 might have initiated from bats, through escaping to intermediate hosts, and behaved as a zoonotic virus [25,26]. Consequently, the zoonotic aspects of SARS-CoV-2 in comparison to SARS-CoV and MERS-CoV are highlighted in this review article.

### SARS-CoV

The causative agent of this syndrome is the SARS-CoV that is characterized by rapidly fatal viral pulmonary pneumonia. SARS first occurred in China between 2002 and 2004, subsequently circulated in Europe and North America due to international travelers. Moreover, the disease was transmitted from human to human. The disease was considered as a zoonotic disease, and its genetic characterization indicated that the insertion of SARS-CoV into the human population occurred from civet cats. Other animals sold in China’s live-animal markets were also considered as a source of transmission of the virus to people [27]. During the SARS outbreaks, there were about 8096 cases from different countries around the globe, accompanied by 774 fatalities (9.6%). Hu *et al*. [28] identified the origin of SARS CoV genetically in the colony of horseshoe bats. They raised the possibility that bats were a source of the virus, before moving to the civet cat or other mammals in the China live-animal markets. Hence, the virus was called a super spreader, because of its transmission between humans through respiratory droplets and close interactions with some individuals. The symptoms of SARS generally appeared in the infected patients within 2-12 days after infection, and the deaths occurred more in the elderly and immunosuppressed individuals. However, the severe form of the disease was not common in children and youngsters [29]. The most common symptoms of SARS were generally not specific. These included malaise and myalgia associated with lymphopenia and thrombocytopenia. Elevation in some enzymes, such as the C-reactive protein and lactate dehydrogenase, has also occurred.

### The MERS-CoV

The MERS is a severe respiratory syndrome caused by MERS-CoV and characterized by a high fatality rate case. The first case of MERS was reported in September 2012, from a Kingdom Saudi Arabia, in a patient suffering from respiratory and renal failure [21]. MERS was continuously spreading within the Arabian Peninsula, as well as in various countries around the world due to the international traveling of the infected people. The total confirmed cases of MERS were 2266, accompanied by 804 fatalities (35.5% case fatality) [20]. The MERS is a zoonotic disease. The introduction of MERS into the human population was through dromedary camels, according to the serological and molecular investigations [30]. Afterward, suspicion had been raised about the role of camels as intermediate hosts or reservoirs, and the studies had found genomic fragmented material of MERS-CoV, identical to the human strains [31]. The first suggestion of MERS-CoV spillover into the human population was from camels. However, nosocomial spreading between people was reported in most cases, such as the outbreak that occurred in the Korean hospital [32].

In addition, virus transmission occurred in households due to contact between people [33]. The clinical signs of MERS were variable and ranged from asymptomatic infection in 25% to severe disease with elevated mortality in the most significant risk groups, including older adults and diabetes and heart disease patients, who were likely to develop respiratory failure [32-34]. The percentage of positive MERS-CoV antibodies from more than 10.000 infected people in the Kingdom of Saudi Arabia was 0.15% of the patients [35]. However, the positive serological result showed a raised probability among individuals with a history of camel exposure. These people might prove to be asymptomatic sources of infection. The most common MERS clinical symptoms are non-specific, including myalgias, sore throat, and runny nose with an incubation period from 2 to 14 days. In addition, extrapulmonary manifestations include gastrointestinal distress. Furthermore, neurological sequelae have been reported in some cases accompanied by respiratory symptoms [32]. Leukopenia, thrombocytopenia, and anemia are the most common laboratory disorders in MERS-infected patients.

### The SARS-CoV 2 (COVID-19)

COVID-19 is considered as the viral disease of the century, which has caused health crisis, fear, and severe distress among the population worldwide. The disease is termed as “severe acute respiratory syndrome-coronavirus-2” and its causative agent had earlier been called “2019-nCoV” and renamed as “SARS-CoV-2.” It is considered a deadly disease, but it can be resolved, with 2% case fatality and diffuse alveolar damage, that may lead to progressive respiratory failure in severe cases of the disease [1,36,37]. COVID-19 was first recognized in December 2019, in 27 human cases (with respiratory signs) in China/Wuhan. In addition, seven patients were seriously ill and had a history of previous exposure to live animals, such as, bats, snakes, and farm animals, at the Huanan seafood market, which was proposed to be the possible origin of zoonosis [26,38]. A novel CoV was recognized from the patients and the virus was termed as a “2019-Novel Coronavirus” (2019-nCoV), which was related to the year of emergence. However, the virus was renamed by the World Health Organization (WHO) in February 2020, as COVID-19-SARS-CoV-2. Contrary to SARS and MERS, the ancestors of the virus, SARS-CoV-2 has infected more people around the world. COVID-19 is recorded in all continents and it is progressively rising in many countries.

On November 19, 2020, about 56,828,218 cases had been confirmed, with over 1,359,320 deaths and 39,548,923 had recovered. The statistics showed 15,919,975 active cases; 15,818,613 (99%) in mild condition; 101,362 (1%) serious or critical; and closed cases comprised 40,908,243, with 39,548,923 (97%) recovered/discharged and 1,359,320 (3%) deaths (https://www.worldometers.info/coronavirus/) [39].

The infection occurred in patients with an average age of 55 years. However, cases appeared to be sporadic in children. The phylogenetic investigation and full genome sequencing indicated that the beta-CoV, SARS-CoV-19, was situated within the SARS-CoV subgenus, together with several bat CoVs, which was the cause of COVID-19. However, this virus was located in a different clade.

Moreover, both viruses owned similar cell entry receptors, the angiotensin-converting enzyme 2 (ACE2) [40]. Two bat CoV RNA sequences were found to be closely similar to COVID-19-COV-2, and bats appeared to be the origin of infection, though its transmission is still mysterious. Hitherto, no one knows whether the virus used some mechanism of transmission, such as, an intermediate host, or was transmitted directly from bats [41]. A phylogenetic study of 103 strains of SARS-CoV-2 isolated from Chinese patients was recognized to be of two different types. They were named “type L” and “type S,” which accounted for 70% and 30% of the strains, respectively. The early days of the epidemic were dominated by the L type. At the same time, it occurred in a lower proportion with virus strains that were isolated from outside rather than inside Wuhan, accompanied by uncertain clinical implications.

The risk of SARS-CoV-2 transmission is still incomplete and needs further understanding. The earlier epidemiological investigation from Wuhan blamed the exposure of the patients to live animals in the seafood market, and the records proved that the patients worked or visited these places. The scenario was one of continuous spreading of the disease due to contact between people. Person-to-person transmission appeared to be the primary method, which occurred principally through respiratory droplets, similar to the spread of influenza. Furthermore, the primary source of infection was felt to be a patient’s respiratory discharge during a sneeze, cough, and talk with other people, particularly the direct exposure of mucous membranes. Touching smooth infected surfaces and touching one’s eyes, nose, and mouth were also considered as an important route of infection [42].

The live SARS-CoV-2 was isolated from patients through various clinical specimens, such as blood and stool [43-45]. However, according to the WHO report, the fecal–oral route did not appear to play a significant role in the transmission of the disease [46].

Hitherto, several facts regarding COVID-19-SARS-CoV-2 are still uncertain, such as the period of infection and the interval time for the appearance of infection in the infected person. Immediately following the advent of symptoms, the levels of the viral RNA seem to be higher compared to the latter period of the illness [47]. Therefore, there is a possibility of higher transmission of the virus in the earlier stage of infection, but more evidence is required to prove this postulation. Furthermore, the severity of the illness has been found to affect the duration of viral shedding. Liu *et al*. [48] studied 21 patients with mild illness. They showed that 10 days after onset of symptoms, repeated negative viral RNA tests were obtained from 90% of the patients, through nasopharyngeal swabs. Nonetheless, more severe illness appeared positive for longer. Even as viral RNA shedding meant the duration of the oropharyngeal specimens was 20 days, ranging from 8 to 37 days, according to a retrospective cohort study of 137 patients, who survived COVID-19 [40].

However, the transmission rates from symptomatic infected individuals varied by location and infection control interventions; while, the extent of occurrence of COVID-19 is still a mystery [49]. The most common clinical signs in COVID-19 patients are high fever, cough, and shortness of breath, accompanied by pneumonia in most severe cases, and 2% fatality. Although COVID-19 has a lower mortality rate than MERS-CoV, more deaths have occurred because of increasing numbers of infected people around the globe.

Therefore, the Chinese authorities have taken the universal threat very seriously, and the suppression measures have been extraordinary such as closing the roads to Wuhan, building a hospital in record time, and also closing of airports and train stations. Nevertheless, a thousand cases with SARS-CoV-2 have already been reported in many nations. High numbers of infected people were reported in the USA, European Union, United Kingdom, Middle East, Iran, Africa, New Zealand, and Australia [37,44,50-52].

### Zoonotic Aspects of COVID-19 (SARS-CoV-2), MERS-CoV, and SARS-CoV

Zoonotic diseases are caused by viral, parasitic, and bacterial germs that circulate between animals and humans. Along with the history of nations, several bugs have emerged in the population that threaten human life and cause fear and health crisis. Some of these diseases are zoonotic in origin, for example, salmonellosis, Ebola virus, and CoV. The zoonotic reservoirs are the origin of most strains of new viruses. CoVs have quickly developed a potential ability to cross species among domestic animals and have given rise to three highly pathogenic human strains, including SARS-CoV, MERS-CoV, and last, SARS-CoV-2 [41].

**Figure-1:**
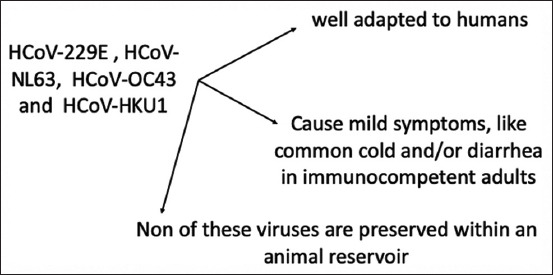
The well-adapted strains of coronaviruses from humans.

Animal CoVs have been recognized since the 1930s; however, some CoVs have adapted well to humans and correlated with diarrhea, common cold, and some mild symptoms in immunocompetent adults and are widely distributed in the human population, but none of these viruses are preserved within the animal reservoir (Figure-1) [53,54]. On the contrary, SARS-CoV, MERS-CoV, and SARS-CoV-2 are related to the animal reservoir and are associated with severe lower respiratory tract infection, with a high ability to develop acute respiratory distress syndrome with extrapulmonary manifestation. Studies have proved that SARS-CoV and MERS-CoV have not adequately acclimatized to humans and are probably distributed chiefly in a zoonotic pool that might need an intermediate host species to spill over the infection to the susceptible human population [12,36,55].

The SARS-CoV outbreak began with the exposure of the first patient to the animal’s reservoirs before the onset of symptoms, suggesting a zoonotic origin to this syndrome. Several attempts have been made to recognize the source of the SARS-CoV infection, depending on virus isolation and molecular and seroepidemiological analysis. They found that masked palm civets (*Paguma larvata*) were the potential source of human infection, as the persons in contact with the same animals were positive for antibodies against SARS-CoV [12].

Later on, no SARS-CoV was recognized from farmed or wild civets in other epidemiological studies. Accordingly, other animals were accused of being the natural reservoirs of SARS-CoV, who circulated the virus to the live animals in the market, where masked palm civets acted as intermediate hosts. Eventually, bats were considered as the natural reservoirs of SARS-CoV. This suggestion was supported by the results of the previous studies on other zoonotic diseases that considered bats as the origin of the Nipah and Hendra viruses[56-60]. Consequently, studies have recognized novel CoVs associated with SARS-CoV in horseshoe bats (*Rhinolophus*) in China. The virus was called the “SARS-like coronavirus” (SL-CoV) [61,62]. The genetic studies of SL-CoV showed 88-90% identity of the genome sequence between themselves, and 87-92% were identical to human or masked palm civet SARS-CoV strains. In addition, an ORF distinctive set was found in both SARS-CoV and bats SL-CoV, confirming the narrow phylogenetic relationship between them.

**Figure-2:**
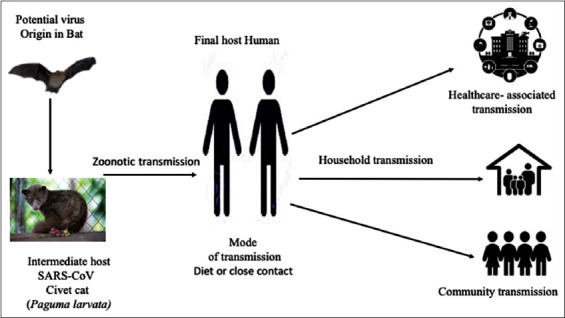
Briefing of severe acute respiratory syndrome coronavirus transmission pathways.

Consequently, the RNA of SL-CoV was isolated from the geographically broader species of bats, the *Rhinolophus*, in China. Scientists recognized the resemblance between SL-CoV and SARS-CoV, genetically. They found that SL-CoV owns the functional S protein and can use a similar ACE2 receptor. All these facts are strong evidence supporting the notion that bats are the origin and the main reservoirs for CoVs, including SARS-CoV (Figure-2). The adaptation of viruses in different species of bats and other animal hosts has also been proved by scientists [12]. However, the gene encoding S protein showed considerable variability in its receptor-binding domain (RBD) that determined viral tropism and host range [12].

Only one bat’s CoV showed a sequence of S protein that permitted the attachment of ACE2 used by SARS-CoV, to damage the lower respiratory tract epithelial cells. Therefore, modification of the S protein requires new spillover events from an animal reservoir to humans, to enable it to infect the cells of the human respiratory system. This is the primary phase to overcome the close barrier. In addition, the viral gene *orf8* can also mutate during viral adaptation to the new host. Consequently, the capacity to initiate new viral variants can encourage a spread from bats to other sylvatic animals, the modification to a new host, and then the transmission to other indigenous animals and human beings [12].

Dromedary camelids were found to be the reservoir host of MERS-CoV. However, this speculation was denied, and bats were proposed as the probable original reservoirs. The scientists have identified strains of CoVs similar to MERS-CoV in bats. At the same time, dromedary camelids were the only species that were implicated to act as intermediate hosts in the transmission of MERS-CoV [63,64].

**Figure-3:**
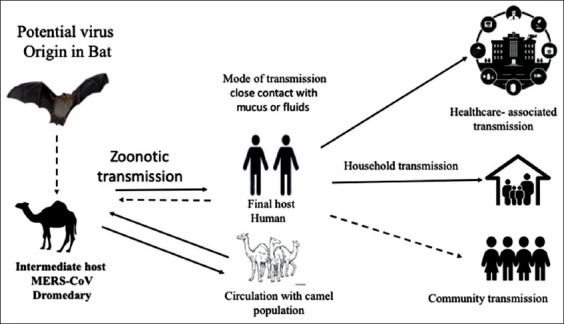
Briefing of Middle East respiratory syndrome-coronavirus transmission routes. Solid lines designate identified transmission pathways and dashed lines designate possible transmission routes for which supporting proof is incomplete or indefinite.

The serological studies on dromedaries, carried out in Oman, Saudi Arabia, United Arab Emirates, Qatar, and Jordan, proved that they had seropositivity neutralizing antibodies for MERS-CoV [65]. While, the middle east’s camels that raised in Nigeria, Egypt, Kenya, Ethiopia, Tunisia, Somalia, and Sudan, were also showed same results[66,67]. Successive investigations in different countries have proved the isolation of live MERS-CoV from camelids. It was isolated mainly from nasal cavities and pointed out that dromedaries are an important source of MERS-CoV infection [68,69]. On the other hand, numerous confirmed infected cases revealed no contact history with camels, proposing the direct transmission between humans (human-to-human spreading), or contact with animal species that retained yet to be identified MERS-CoV (Figure-3) [70,71]. The molecular investigation proved that the bat CoV (HKU4) was more phylogenetically and closely related to the MERS-CoV.

In addition, this strain could also utilize the virus entry receptor, the CD26 [72]. Therefore, the MERS-CoV was proposed as a zoonotic disease that originated from bats and showed similarities with HKU4, where both viruses have the CD26 receptor. Although, scientists did not isolate MERS-CoV from wild bats and also no viral sequences have been identified [73]. In addition, phylogenetic analyses displayed a gap between bat CoVs and the MERS-CoVs, suggesting the existence of other CoVs that have not been identified yet [12].

The current CoV pandemic is caused by SARS-COV-2 that has produced severe health crises and public health disasters worldwide. Initially, the causative virus was speculated to have originated from a specific species of snakes sold in the live-animal market in Wuhan/China, such as *Bungarus multicinctus* or *Naja atra* (Chinese cobra). However, this speculation has been ignored. According to the knowledge obtained from the previous SARS-CoV and MERS-CoV outbreaks, all RNA viruses have the following features, high mutation rates, ease of rapid transmission from one species to another (cross-species), and their evolving nature [74]. However, ancestral CoVs harbored by bats are considered as the origin of CoVs, while other animals act as intermediate hosts, and humans serve as the final hosts. Several risk factors play a role in the initiation of a new, novel, human CoV. These include close communication between humans and live animals and the habit of consumption of uncooked meat. Scientists believe that SARS-CoV-2 originated from the animal and seafood market in Wuhan/China. These places are engaged in selling local and exotic animals and their parts, which are habitually used in food or traditional medicine, such as, the scales of pangolin and tiger paws, used in Traditional Chinese medicine. Similar to SARS, COVID-19 is deemed to be a zoonotic disease that primarily spills over from animals to humans, although transmission from humans to humans has been proved and has become a major source of infection. The genetic studies on the virus isolated from human viral pneumonia in the Wuhan outbreak have investigated a new CoV (*Betacoronavirus*) that is sufficiently different from the SARS-CoV. The virus has been named “2019-nCoV” and later renamed as “SARS-CoV-2.”

The scientists proposed that the bat was the original reservoir of this virus. At the same time, the other animals in the live-animal markets served as intermediate hosts that hastened the emergence of the virus in human beings. Studies proved that the SARS-CoV-2 genomes differed about <0.1% (more than 99.98% of sequence identity) during structural analysis. Moreover, the virus was capable of attaching and binding the ACE 2 human receptor. However, the continuity of viral spreading led to raising more mutations, which increased the virulence of the virus. Different species of bats were considered as natural reservoir hosts for a number of CoVs. They were also identified to harbor a large number of SARS-related CoVs (SARSr-CoVs) [28,61,75]. It is worth mentioning that some bat SARSr-CoVs were able to infect humans [40,76,77]. The full-length genome sequences of SARS-CoV-2 had been characterized from the initial stage of COVID-19, in five patients, from Wuhan, China [26]. The identity of the virus sequences appeared nearly and share about 79.6% of SARS-CoV. Furthermore, SARS-CoV-2 was identical, about 96%, to the whole genome level of the bat CoV. Furthermore, the virus appeared to belong to the SARSr-CoV by sequence analysis. In severely and critically ill patients, the SARS-CoV-2 isolated from the bronchoalveolar lavage was neutralized by the sera of several SARS-CoV patients. These findings proved the similarity between the SARS-CoV and SARS-CoV-2 in the cell entry receptor, the ACE2.

**Figure-4:**
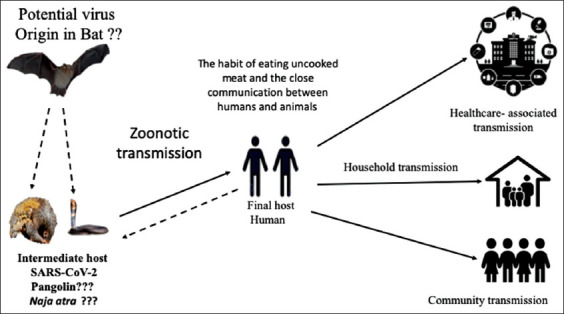
Briefing of severe acute respiratory syndrome coronavirus-2 transmission routes. The solid lines designate identified transmission pathways and the dashed lines designate the possible transmission routes for which supporting proof is incomplete or indefinite.

The studies also proved that 96.2% of SARS-CoV-2 nucleotide appeared to share the homology of the bats’ CoV RaTG13, which was identified in the bat species *Rhinolophus affinis*. In addition, it presumed that the bat was not the direct reservoir host of SARS-CoV-2, if no identical bat CoVs were found in the future. Many doubts remain on the actual intermediate hosts of SARS-CoV-2. The wildlife species and killed animals sold in the Huanan seafood wholesale market were taken as the possible option, as the majority of the initial COVID-19 patients had a history of previous exposure to these areas, indicating the zoonotic nature of the disease, with the possibility of a human-to-human transmission experience [37]. Metagenomic sequencing studies of the virus found a group of small, rare vertebrates called pangolins (*Manis javanica*) (Figure-4) that harbor a close relation to the ancestral beta-CoVs and SARS-CoV-2. The identified pangolin’s CoV shared 85-92% nucleotide sequence homology with SARS-CoV-2. However, another genomic study showed about 90% identity at the level of nucleotide sequences that were closely related and similar to RaTG13. The phylogenetic tree showed that these virus groups were located in the two sublineages of the SARS-CoV-2-like viruses. Furthermore, these viruses share a similar RBD with SARS-CoV-2, with about 97.4% amino acid sequence identity [78]. Still, scientists accept the theory that the pangolin is one of the possible intermediate hosts that transmit SARS-CoV-2, although there is the absence of a direct indication that supports pangolin source of SARS-CoV-2, because of the divergence between the sequences of SARS-CoV-2 and pangolin SARS-CoV-2-related beta-CoVs. SARS-CoV-2 reveals a shorter gap with RaTG13 than with pangolin SARS-CoV-2-related beta-CoVs. Therefore, COVID-19 is considered as being the immediate zoonotic origin. Furthermore, the evolving route of SARS-CoV-2 in bats, pangolins, and other mammals still needs to be verified. Moreover, a third wild animal species might be presumed to hold the recombination between a pangolin SARS-CoV-2-related beta-CoV and RaTG13, as the evolution driving force and recombination are widely spread between beta-CoVs.

## Conclusion

Three zoonotic CoV outbreaks have emerged during the past two decades. These diseases are SARS, MERS, and COVID-19, which were caused by SARS-CoV, MERS-CoV, and SARS-CoV-2, respectively. COVID-19 is the most recent pandemic disease. It has spread rapidly worldwide and caused fatalities due to acute respiratory disease. COVID-19 has created panic and severe fear among people, and has raised extensive social, economic, and health crises, accompanied by precautionary procedures that have been applied by a majority of countries to contain this virus transmission. COVID-19 has altered the opinion on the significance of zoonotic diseases and their animal origins and intermediate reservoirs, which may be some other wild animals. Genetically, the CoV is derived from the SARS-like bat CoVs. However, the virus is less related to the MERS-CoV. The SARS-CoV-2 uses the same RBD receptor (in the Spike protein) of SARS-CoV – the ACE2. Conversely, DPP4 (CD26), the MERS-CoVs primary receptor, has not been proved to be the receptor of SARS-CoV-2.

## Authors’ Contributions

KAA suggested the concept of the study, wrote the major line, drafted, and critical checking of this manuscript. JMK was contributed in writing, preparing, and final reviewing of this manuscript. Both authors read and approved the final manuscript.

## References

[1] Biscayart C, Angeleri P, Lloveras S, Chaves T.D, Schlagenhauf P, Rodríguez-Morales A.J (2020). The next big threat to global health?2019 novel coronavirus (2019-nCoV): What advice can we give to travellers?Interim recommendations January 2020, from the Latin-American Society for Travel Medicine (SLAMVI). Travel Med. Infect. Dis.

[2] Durham P.J, Stevenson B.J, Farquharson B.C (1979). Rotavirus and coronavirus associated diarrhea in domestic animals. N. Z. Vet. J.

[3] Weiss S.R, Leibowitz J.L (2011). Coronavirus pathogenesis. Adv. Virus Res.

[4] Masters P.S, Perlman S, Knipe D.M, Howley P.M (2013). Coronaviridae. Fields Virology.

[5] Fehr A.R, Perlman S (2015). Coronaviruses: An overview of their replication and pathogenesis. Methods Mol. Biol.

[6] Perlman S, Netland J (2009). Coronaviruses post-SARS:Update on replication and pathogenesis. Nat. Rev. Microbiol.

[7] Lai M, Zhou J, Knipe D.M, Howley P.M (2007). Coronaviridae. Fields Virology.

[8] Lai M.M, Cavanagh D (1997). The molecular biology of coronaviruses. Adv. Virus Res.

[9] Tang X, Wu C, Li X, Song Y, Yao X, Wu X, Duan Y, Zhang H, Wang Y, Qian Z, Cui J, Lu J (2020). On the origin and continuing evolution of SARS-CoV-2. Natl. Sci. Rev.

[10] Langereis M.A, van Vliet A.L.W, Boot W, de Groot R.J (2010). Attachment of mouse hepatitis virus to O-acetylated sialic acid is mediated by hemagglutinin-esterase and not by the spike protein. J. Virol.

[11] Channappanavar R, Perlman S (2017). Pathogenic human coronavirus infections:causes and consequences of cytokine storm and immunopathology. Semin. Immunopathol.

[12] Cui J, Li F, Shi Z.L (2019). Origin and evolution of pathogenic coronaviruses. Nat. Rev. Microbiol.

[13] El-Kafrawy S.A, Corman V.M, Tolah A.M, Al Masaudi S.B, Hassan A.M, Müller M.A, Bleicker T, Harakeh S.M, Alzahrani A.A, Alsaaidi G.A, Alagili A.N, Hashem A.M, Zumla A, Drosten C, Azhar E.I (2019). Enzootic patterns of Middle East respiratory syndrome coronavirus in imported African and local Arabian dromedary camels: A prospective genomic study. Lancet Planet. Health.

[14] Oh M.D, Park W.B, Park S.W, Choe P.G, Bang J.H, Song K.H, Kim E.S, Kim H.B, Kim N.J (2018). Middle East respiratory syndrome:What we learned from the 2015 outbreak in the Republic of Korea. Korean J. Intern. Med.

[15] Kim K.H, Tandi T.E, Choi J.W, Moon J.M, Kim M.S (2017). Middle East respiratory syndrome coronavirus (MERS-CoV) outbreak in South Korea, 2015: Epidemiology, characteristics and public health implications. J. Hosp. Infect.

[16] Zumla A, Hui D.S, Perlman S (2015). Middle East respiratory syndrome. Lancet (London, England).

[17] Drosten C, Kellam P, Memish Z.A (2014). Evidence for camel-to-human transmission of MERS coronavirus. N. Engl. J. Med.

[18] Peiris J.S, Yuen K.Y, Osterhaus A.D, Sto?hr K (2003). The severe acute respiratory syndrome. N. Engl. J. Med.

[19] https://apps.who.int/iris/bitstream/handle/10665/70863/WHO_CDS_CSR_GAR_2003.11_eng.pdf.

[20] https://www.who.int/emergencies/diseases/novel-coronavirus-2019?gclid=Cj0KCQiA6Or_BRC_ARIsAPzuer_iVN0arBnRgFPngDy4geZBVu1rHLblqyOq55luedGsZ9zzBShaagsaAvbFEALw_wcB.

[21] Zaki A.M, van Boheemen S, Bestebroer T.M, Osterhaus A.D, Fouchier R.A (2012). Isolation of a novel coronavirus from a man with pneumonia in Saudi Arabia. N. Engl. J. Med.

[22] Zhu N, Zhang D, Wang W, Li X, Yang B, Song J, Zhao X, Huang B, Shi W, Lu R, Niu P, Zhan F, Ma X, Wang D, Xu W, Wu G, Gao G.F, Tan W, China Novel Coronavirus Investigating and Research Team (2020). A novel coronavirus from patients with pneumonia in China, 2019. N. Engl. J. Med.

[23] Nishiura H, Jung S.M, Linton N.M, Kinoshita R, Yang Y, Hayashi K, Kobayashi T, Yuan B, Akhmetzhanov A.R (2020). The extent of transmission of novel coronavirus in Wuhan, China, 2020. J. Clin. Med.

[24] Ji W, Wang W, Zhao X, Zai J, Li X (2020). Cross-species transmission of the newly identified coronavirus 2019-nCoV. J. Med. Virol.

[25] Paraskevis D, Kostaki E.G, Magiorkinis G, Panayiotakopoulos G, Sourvinos G, Tsiodras S (2020). Full-genome evolutionary analysis of the novel coronavirus (2019-nCoV) rejects the hypothesis of emergence as a result of a recent recombination event. Infect. Genet. Evol.

[26] Zhou G, Chen S, Chen Z (2020). Advances in COVID-19: The virus, the pathogenesis, and evidence-based control and therapeutic strategies. Front. Med.

[27] Guan Y, Zheng B.J, He Y.Q, Liu X.L, Zhuang Z.X, Cheung C.L, Luo S.W, Li P.H, Zhang L.J, Guan Y.J, Butt K.M, Wong K.L, Chan K.W, Lim W, Shortridge K.F, Yuen K.Y, Peiris J.SM, Poon L.L.M (2003). Isolation and characterization of viruses related to the SARS coronavirus from animals in Southern China. Science.

[28] Hu B, Zeng L.P, Yang X.L, Ge X.Y, Zhang W, Li B, Xie J.Z, Shen X.R, Zhang Y.Z, Wang N, Luo D.S, Zheng X.S, Wang M.N, Daszak P, Wang L.F, Cui J, Shi Z.L (2017). Discovery of a rich gene pool of bat SARS-related coronaviruses provides new insights into the origin of SARS coronavirus. PLoS Pathog.

[29] Chan J.C, Tsui E.L, Wong V.C, Hospital Authority SARS Collaborative Group (2007). Prognostication in severe acute respiratory syndrome: A retrospective time-course analysis of 1312 laboratory-confirmed patients in Hong Kong. Respirology.

[30] Azhar E.I, El-Kafrawy S.A, Farraj S.A, Hassan A.M, Al-Saeed M.S, Hashem A.M, Madani T.A (2014). Evidence for camel-to-human transmission of MERS coronavirus. N. Engl. J. Med.

[31] Memish Z.A, Mishra N, Olival K.J, Fagbo S.F, Kapoor V, Epstein J.H, Alhakeem R, Durosinloun A, Al Asmari M, Islam A, Kapoor A, Briese T, Daszak P, Al Rabeeah A.A, Lipkin W.I (2013). Middle East respiratory syndrome coronavirus in bats, Saudi Arabia. Emerg. Infect. Dis.

[32] Arabi Y.M, Balkhy H.H, Hayden F.G, Bouchama A, Luke T, Baillie J.K, Al-Omari A, Hajeer A.H, Senga M, Denison M.R, Nguyen-Van-Tam J.S, Shindo N, Bermingham A, Chappell J.D, Van Kerkhove M.D, Fowler R.A (2017). Middle east respiratory syndrome. N. Engl. J. Med.

[33] Arwady M.A, Alraddadi B, Basler C, Azhar E.I, Abuelzein E, Sindy A.I, Sadiq B.M, Althaqafi A.O, Shabouni O, Banjar A, Haynes L.M, Gerber S.I, Feikin D.R, Madani T.A (2016). Middle East respiratory syndrome coronavirus transmission in extended family, Saudi Arabia, 2014. Emerg. Infect. Dis.

[34] Oboho I.K, Tomczyk S.M, Al-Asmari A.M, Banjar A.A, Al-Mugti H, Aloraini M.S, Alkhaldi K.Z, Almohammadi E.L, Alraddadi B.M, Gerber S.I, Swerdlow D.L, Watson J.T, Madani T.A (2015). 2014 MERS-CoV outbreak in Jeddah--a link to health care facilities. N. Engl. J. Med.

[35] Müller M.A, Meyer B, Corman V.M, Al-Masri M, Turkestani A, Ritz D, Sieberg A, Aldabbagh S, Bosch B.J, Lattwein E, Alhakeem R.F, Assiri A.M, Albarrak A.M, Al-Shangiti A.M, Al-Tawfiq J.A, Wikramaratna P, Alrabeeah A.A, Drosten C, Memish Z.A (2015). Presence of Middle East respiratory syndrome coronavirus antibodies in Saudi Arabia: A nationwide, cross-sectional, serological study. Lancet Infect. Dis.

[36] Chan J.F, Kok K.H, Zhu Z, Chu H, To K.K, Yuan S, Yuen K.Y (2020). Genomic characterization of the 2019 novel human-pathogenic coronavirus isolated from a patient with atypical pneumonia after visiting Wuhan. Emerg. Microbes. Infect.

[37] Huang C, Wang Y, Li X, Ren L, Zhao J, Hu Y, Zhang L, Fan G, Xu J, Gu X, Cheng Z, Yu T, Xia J, Wei Y, Wu W, Xie X, Yin W, Li H, Liu M, Xiao Y, Gao H, Guo L, Xie J, Wang G, Jiang R, Gao Z, Jin Q, Wang J, Cao B (2020). Clinical features of patients infected with 2019 novel coronavirus in Wuhan, China. Lancet.

[38] Lu H, Stratton C.W, Tang Y.W (2020). Outbreak of pneumonia of unknown etiology in Wuhan, China: The mystery and the miracle. J. Med. Virol.

[39] https://www.worldometers.info/coronavirus/.

[40] Zhou F, Yu T, Du R, Fan G, Liu Y, Liu Z, Xiang J, Wang Y, Song B, Gu X, Guan L, Wei Y, Li H, Wu X, Xu J, Tu S, Zhang Y, Chen H, Cao B (2020). Clinical course and risk factors for mortality of adult inpatients with COVID-19 in Wuhan, China: A retrospective cohort study. Lancet.

[41] Perlman S (2020). Another decade, another coronavirus. N. Engl. J. Med.

[42] https://www.who.int/docs/default-source/coronaviruse/situation-reports/20200122-sitrep-2-2019-ncov.pdf.

[43] Centers for Disease Control and Prevention (2019). Interim Clinical Guidance for Management of Patients with Confirmed 2019 Novel Coronavirus (2019-nCoV) Infection. https://www.cdc.gov/coronavirus/2019-ncov/hcp/clinical-guidance-management-patients.html.

[44] Wang W, Xu Y, Gao R, Lu R, Han K, Wu G, Tan W (2020). Detection of SARS-CoV-2 in different types of clinical specimens. JAMA.

[45] Chen W, Lan Y, Yuan X, Deng X, Li Y, Cai X, Li L, He R, Tan Y, Deng X, Gao M, Tang G, Zhao L, Wang J, Fan Q, Wen C, Tong Y, Tang Y, Hu F, Li F, Tang X (2020). Detectable 2019-nCoV viral RNA in blood is a strong indicator for the further clinical severity. Emerg. Microbes. Infect.

[46] http://www.who.int/docs/default-source/coronaviruse/who-china-joint-mission-on-covid-19-final-report.pdf.

[47] Zou L, Ruan F, Huang M, Liang L, Huang H, Hong Z, Yu J, Kang M, Song Y, Xia J, Guo Q, Song T, He J, Yen H.L, Peiris M, Wu J (2020). SARS-CoV-2 viral load in upper respiratory specimens of infected patients. N. Engl. J. Med.

[48] Liu Y, Yan L.M, Wan L, Xiang T.X, Le A, Liu J.M, Peiris M, Poon L.L.M, Zhang W (2020). Viral dynamics in mild and severe cases of COVID-19. Lancet Infect. Dis.

[49] Rothe C, Schunk M, Sothmann P, Bretzel G, Froeschl G, Wallrauch C, Zimmer T, Thiel V, Janke C, Guggemos W, Seilmaier M, Drosten C, Vollmar P, Zwirglmaier K, Zange S, Wölfel R, Hoelscher M (2020). Transmission of 2019-nCoV infection from an asymptomatic contact in Germany. N. Engl. J. Med.

[50] Yang X, Yu Y, Xu J, Shu H, Xia J, Liu H, Wu Y, Zhang L, Yu Z, Fang M, Yu T, Wang Y, Pan S, Zou X, Yuan S, Shang Y (2020). Clinical course and outcomes of critically ill patients with SARS-CoV-2 pneumonia in Wuhan, China: A single-centered, retrospective, observational study. Lancet Respir. Med.

[51] He S, Han X, Jiang N, Cao Y, Alwalid O, Gu J, Fan Y, Zheng C (2020). Radiological findings from 81 patients with COVID-19 pneumonia in Wuhan, China: A descriptive study. Lancet.

[52] Wang D, Hu B, Hu C, Zhu F, Liu X, Zhang J, Wang B, Xiang H, Cheng Z, Xiong Y, Zhao Y, Li Y, Wang X, Peng Z (2020). Clinical characteristics of 138 hospitalized patients with 2019 novel coronavirus-infected pneumonia in Wuhan, China. JAMA.

[53] Bucknall R.A, King L.M, Kapikian A.Z, Chanock R.M (1972). Studies with human coronaviruses II. Some properties of strains 229E and OC43. Proc. Soc. Exp. Biol. Med.

[54] Woo P.C, Lau S.K, Li K.S, Poon R.W, Wong B.H, Tsoi H.W, Yip B.C, Huang Y, Chan K.H, Yuen K.Y (2006). Molecular diversity of coronaviruses in bats. Virology.

[55] Su S, Wong G, Shi W, Liu J, Lai A.C.K, Zhou J, Liu W, Bi Y, Gao G.F (2016). Epidemiology, genetic recombination, and pathogenesis of coronaviruses. Trends Microbiol.

[56] Kan B, Wang M, Jing H, Xu H, Jiang X, Yan M, Liang W, Zheng H, Wan K, Liu Q, Cui B, Xu Y, Zhang E, Wang H, Ye J, Li G, Li M, Cui Z, Qi X, Chen K, Du L, Gao K, Zhao Y.T, Zou X.Z, Feng Y.J, Gao Y.F, Hai R, Yu D, Guan Y, Xu J (2005). Molecular evolution analysis and geographic investigation of severe acute respiratory syndrome coronavirus-like virus in palm civets at an animal market and on farms. J. Virol.

[57] Poon L.L, Chu D.K, Chan K.H, Wong O.K, Ellis T.M, Leung Y.H, Lau S.K, Woo P.C, Suen K.Y, Yuen K.Y, Guan Y, Peiris J.S (2005). Identification of a novel coronavirus in bats. J. Virol.

[58] Wu D, Tu C, Xin C, Xuan H, Meng Q, Liu Y, Yu Y, Guan Y, Jiang Y, Yin X, Crameri G, Wang M, Li C, Liu S, Liao M, Feng L, Xiang H, Sun J, Chen J, Sun Y, Gu S, Liu N, Fu D, Eaton B.T, Wang L.F, Kong X (2005). Civets are equally susceptible to experimental infection by two different severe acute respiratory syndrome coronavirus isolates. J. Virol.

[59] Yob J.M, Field H, Rashdi A.M, Morrissy C, van der Heide B, Rota P, bin Adzhar A, White J, Daniels P, Jamaluddin A, Ksiazek T (2001). Nipah virus infection in bats (order *Chiroptera*) in peninsular Malaysia. Emerg. Infect. Dis.

[60] Halpin K, Young P.L, Field H.E, Mackenzie J.S (2000). Isolation of Hendra virus from pteropid bats: A natural reservoir of Hendra virus. J. Gen. Virol.

[61] Li W, Shi Z, Yu M, Ren W, Smith C, Epstein J.H, Wang H, Crameri G, Hu Z, Zhang H, Zhang J, McEachern J, Field H, Daszak P, Eaton B.T, Zhang S, Wang L.F (2005). Bats are natural reservoirs of SARS-like coronaviruses. Science.

[62] Lau S.K, Woo P.C, Li K.S, Huang Y, Tsoi H.W, Wong B.H, Wong S.S, Leung S.Y, Chan K.H, Yuen K.Y (2005). Severe acute respiratory syndrome coronavirus-like virus in Chinese horseshoe bats. Proc. Natl. Acad. Sci. U. S. A.

[63] Adney D.R, Brown V.R, Porter S.M, Bielefeldt-Ohmann H, Hartwig A.E, Bowen R.A (2016). Inoculation of goats, sheep, and horses with MERS-CoV does not result in productive viral shedding. Viruses.

[64] Corman V.M, Ithete N.L, Richards L.R, Schoeman M.C, Preiser W, Drosten C, Drexler J.F (2014). Rooting the phylogenetic tree of middle East respiratory syndrome coronavirus by characterization of a conspecific virus from an African bat. J. Virol.

[65] Hijawi B, Abdallat M, Sayaydeh A, Alqasrawi S, Haddadin A, Jaarour N, Alsheikh S, Alsanouri T (2013). Novel coronavirus infections in Jordan, April 2012: Epidemiological findings from a retrospective investigation. East Mediterr. Health J.

[66] Reusken C.B, Haagmans B.L, Müller M.A, Gutierrez C, Godeke G.J, Meyer B, Muth D, Raj V.S, Vries L.S, Corman V.M, Drexler J.F, Smits S.L, El Tahir Y.E, De Sousa R, van Beek J, Nowotny N, van Maanen K, Hidalgo-Hermoso E, Bosch B.J, Rottier P, Osterhaus A, Gortázar-Schmidt C, Drosten C, Koopmans M.P (2013). Middle East respiratory syndrome coronavirus neutralising serum antibodies in dromedary camels: A comparative serological study. Lancet Infect. Dis.

[67] Chan J.F, Lau S.K, To K.K, Cheng V.C, Woo P.C, Yuen K.Y (2015). Middle East respiratory syndrome coronavirus:Another zoonotic betacoronavirus causing SARS-like disease. Clin. Microbiol. Rev.

[68] Hemida M.G, Chu D.K, Poon L.L, Perera R.A, Alhammadi M.A, Ng H.Y, Siu L.Y, Guan Y, Alnaeem A, Peiris M (2014). MERS coronavirus in dromedary camel herd, Saudi Arabia. Emerg. Infect. Dis.

[69] Chu D.K, Poon L.L, Gomaa M.M, Shehata M.M, Perera R.A, Abu Zeid D, El Rifay A.S, Siu L.Y, Guan Y, Webby R.J, Ali M.A, Peiris M, Kayali G (2014). MERS coronaviruses in dromedary camels. Egypt. Emerg. Infect. Dis.

[70] Samara E.M, Abdoun K.A (2014). Concerns about misinterpretation of recent scientific data implicating dromedary camels in epidemiology of Middle East respiratory syndrome (MERS). mBio.

[71] Karima A.S (2018). Invited review:Camelids zoonotic diseases. J. Camelid Sci.

[72] Wang Q, Qi J, Yuan Y, Xuan Y, Han P, Wan Y, Ji W, Li Y, Wu Y, Wang J, Iwamoto A, Woo P.C, Yuen K.Y, Yan J, Lu G, Gao G.F (2014). Bat origins of MERS-CoV supported by bat coronavirus HKU4 usage of human receptor CD26. Cell Host Microbe.

[73] Lu G, Hu Y, Wang Q, Qi J, Gao F, Li Y, Zhang Y, Zhang W, Yuan Y, Bao J, Zhang B, Shi Y, Yan J, Gao G.F (2013). Molecular basis of binding between novel human coronavirus MERS-CoV and its receptor CD26. Nature.

[74] Dolan P.T, Whitfield Z.J, Andino R (2018). Mechanisms and concepts in RNA virus population dynamics and evolution. Annu. Rev. Virol.

[75] Ge X.Y, Li J.L, Yang X.L, Chmura A.A, Zhu G, Epstein J.H, Mazet J.K, Hu B, Zhang W, Peng C, Zhang Y.J, Luo C.M, Tan B, Wang N, Zhu Y, Crameri G, Zhang S.Y, Wang L.F, Daszak P, Shi Z.L (2013). Isolation and characterization of a bat SARS-like coronavirus that uses the ACE2 receptor. Nature.

[76] Menachery V.D, Yount B.L. Jr, Debbink K, Agnihothram S, Gralinski L.E, Plante J.A, Graham R.L, Scobey T, Ge X.Y, Donaldson E.F, Randell S.H, Lanzavecchia A, Marasco W.A, Shi Z.L, Baric R.S (2016). A SARS-like cluster of circulating bat coronaviruses shows potential for human emergence. Nat. Med.

[77] Menachery V.D, Yount B.L, Sims A.C, Debbink K, Agnihothram S.S, Gralinski L.E, Graham R.L, Scobey T, Plante J.A, Royal S.R, Swanstrom J, Sheahan T.P, Pickles R.J, Corti D, Randell S.H, Lanzavecchia A, Marasco W.A, Baric R.S (2016). SARS-like WIV1-CoV poised for human emergence. Proc. Natl. Acad. Sci. U. S. A.

[78] Lam T.T, Jia N, Zhang Y.W, Shum M.H, Jiang J.F, Zhu H.C, Tong Y.G, Shi Y.X, Ni X.B, Liao Y.S, Li W.J, Jiang B.G, Wei W, Yuan T.T, Zheng K, Cui X.M, Li J, Pei G.Q, Qiang X, Cheung W.Y, Li L.F, Sun F.F, Qin S, Huang J.C, Leung G.M, Holmes E.C, Hu Y.L, Guan Y, Cao W.C (2020). Identifying SARS-CoV-2-related coronaviruses in Malayan pangolins. Nature.

